# Supplementary material for: MicroCT-based phenomics in the zebrafish skeleton reveals virtues of deep phenotyping in a distributed organ system
Source: eLife. 2017 Sep 8;6:e26014. doi: 10.7554/eLife.26014 (PMC5606849; doi:10.7554/eLife.26014)
Supplement: Figure 4—source data 2. — For these studies we tested two subsets of 8 vertebrae (Group A: Vert 1, 3, 5, 7, 9, 11, 13, and 15; Group B: and Vert 2, 4, 6, 8, 10, 12, 14, and 16). Cent.Th, Neur.Th, Haem.Th.sd, and Cent.Le were all associated with p<0.05 for one or both groups, and thus these phenotypic features were excluded from Monte Carlo simulations. [file elife-26014-fig4-data2.docx]

|  | Group A  p-values | Group B  p-values |
| --- | --- | --- |
| Tot.Vol  Cent.Vol  Haem.Vol  Neur.Vol  Tot.SA  Cent.SA  Haem.SA  Neur.SA  Tot.TMD  Cent.TMD  Haem.TMD  Neur.TMD  Tot.TMD.sd  Cent.TMD.sd  Haem.TMD.sd  Neur.TMD.sd  Tot.Th  Cent.Th  Haem.Th  Neur.Th  Tot.Th.sd  Cent.Th.sd  Haem.Th.sd  Neur.Th.sd  Cent.Le | 0.57  0.21  0.13  0.74  0.61  0.27  0.26  0.71  0.43  0.38  0.58  0.76  0.39  0.45  0.45  0.13  0.30  0.11  0.09  0.20  0.21  0.36  0.09  0.13  <0.01 | 0.43  0.75  0.73  1.00  0.46  0.72  0.72  1.00  0.48  0.38  0.35  0.52  0.30  0.33  0.07  0.53  0.12  0.03  0.63  0.01  0.55  0.27  0.02  0.07  <0.001 |
