# Supplementary material for: MicroCT-based phenomics in the zebrafish skeleton reveals virtues of deep phenotyping in a distributed organ system
Source: eLife. 2017 Sep 8;6:e26014. doi: 10.7554/eLife.26014 (PMC5606849; doi:10.7554/eLife.26014)
Supplement: Figure 4—source data 3. — The top table shows results assuming each phenotypic feature is uniformly elevated in simulated mutants (characteristic effect size: d = 4; sample size: n = 3; alpha = 0.05). The bottom table shows results assuming a non-uniform elevation, with the characteristic effect size linearly increasing from d = 0 at vert 1 to d = 4 at vert 16. [file elife-26014-fig4-data3.docx]

| **Uniform** | | | | | | |
| --- | --- | --- | --- | --- | --- | --- |
|  |  | **Sensitivity** |  |  | **Specificity** |  |
|  | global test, vertebrae 1:16 | t.test, vertebra 2 | t.test, mean of vertebrae 1:16 | global test, vertebrae 1:16 | t.test, vertebra 2 | t.test, mean of vertebrae 1:16 |
| Tot.Vol | 0.97 | 0.89 | 0.94 | 0.95 | 0.97 | 0.96 |
| Cent.Vol | 0.97 | 0.88 | 0.92 | 0.95 | 0.96 | 0.97 |
| Haem.Vol | 0.98 | 0.89 | 0.95 | 0.95 | 0.96 | 0.97 |
| Neur.Vol | 1.00 | 0.88 | 0.99 | 0.95 | 0.97 | 0.97 |
| Tot.SA | 0.97 | 0.88 | 0.93 | 0.95 | 0.97 | 0.97 |
| Cent.SA | 0.97 | 0.88 | 0.92 | 0.95 | 0.96 | 0.96 |
| Haem.SA | 0.98 | 0.89 | 0.95 | 0.95 | 0.97 | 0.97 |
| Neur.SA | 1.00 | 0.89 | 0.99 | 0.95 | 0.97 | 0.96 |
| Tot.TMD | 0.96 | 0.88 | 0.91 | 0.95 | 0.97 | 0.97 |
| Cent.TMD | 0.96 | 0.88 | 0.90 | 0.95 | 0.97 | 0.96 |
| Haem.TMD | 0.98 | 0.89 | 0.93 | 0.95 | 0.97 | 0.96 |
| Neur.TMD | 0.97 | 0.88 | 0.92 | 0.95 | 0.96 | 0.96 |
| Tot.TMD.sd | 0.99 | 0.89 | 0.97 | 0.95 | 0.97 | 0.97 |
| Cent.TMD.sd | 0.99 | 0.89 | 0.96 | 0.95 | 0.97 | 0.97 |
| Haem.TMD.sd | 1.00 | 0.89 | 1.00 | 0.95 | 0.97 | 0.96 |
| Neur.TMD.sd | 1.00 | 0.88 | 0.99 | 0.95 | 0.97 | 0.96 |
| Tot.Th | 0.99 | 0.89 | 0.97 | 0.95 | 0.97 | 0.97 |
| Haem.Th | 1.00 | 0.88 | 1.00 | 0.95 | 0.97 | 0.96 |
| Tot.Th.sd | 1.00 | 0.88 | 1.00 | 0.95 | 0.97 | 0.97 |
| Cent.Th.sd | 1.00 | 0.89 | 1.00 | 0.95 | 0.96 | 0.96 |
| Neur.Th.sd | 1.00 | 0.88 | 1.00 | 0.95 | 0.96 | 0.96 |

| **High Posterior** | | | | | | |
| --- | --- | --- | --- | --- | --- | --- |
|  |  | **Sensitivity** |  |  | **Specificity** |  |
|  | global test, vertebrae 1:16 | t.test, vertebra 2 | t.test, mean of vertebrae 1:16 | global test, vertebrae 1:16 | t.test, vertebra 2 | t.test, mean of vertebrae 1:16 |
| Tot.Vol | 0.50 | 0.04 | 0.29 | 0.95 | 0.96 | 0.96 |
| Cent.Vol | 0.66 | 0.04 | 0.35 | 0.95 | 0.96 | 0.96 |
| Haem.Vol | 0.32 | 0.04 | 0.21 | 0.95 | 0.96 | 0.97 |
| Neur.Vol | 0.74 | 0.04 | 0.45 | 0.95 | 0.97 | 0.97 |
| Tot.SA | 0.51 | 0.04 | 0.29 | 0.95 | 0.97 | 0.97 |
| Cent.SA | 0.71 | 0.04 | 0.38 | 0.95 | 0.97 | 0.96 |
| Haem.SA | 0.34 | 0.04 | 0.22 | 0.95 | 0.96 | 0.96 |
| Neur.SA | 0.75 | 0.04 | 0.46 | 0.95 | 0.96 | 0.96 |
| Tot.TMD | 0.74 | 0.04 | 0.40 | 0.95 | 0.97 | 0.97 |
| Cent.TMD | 0.71 | 0.04 | 0.38 | 0.95 | 0.97 | 0.97 |
| Haem.TMD | 0.84 | 0.04 | 0.48 | 0.95 | 0.96 | 0.96 |
| Neur.TMD | 0.81 | 0.04 | 0.45 | 0.95 | 0.97 | 0.97 |
| Tot.TMD.sd | 0.84 | 0.04 | 0.55 | 0.95 | 0.96 | 0.96 |
| Cent.TMD.sd | 0.81 | 0.04 | 0.50 | 0.95 | 0.96 | 0.97 |
| Haem.TMD.sd | 0.97 | 0.04 | 0.79 | 0.94 | 0.96 | 0.96 |
| Neur.TMD.sd | 0.92 | 0.04 | 0.64 | 0.95 | 0.96 | 0.97 |
| Tot.Th | 0.88 | 0.04 | 0.59 | 0.95 | 0.97 | 0.97 |
| Haem.Th | 0.97 | 0.04 | 0.90 | 0.95 | 0.97 | 0.97 |
| Tot.Th.sd | 0.94 | 0.04 | 0.73 | 0.95 | 0.96 | 0.96 |
| Cent.Th.sd | 0.93 | 0.04 | 0.76 | 0.95 | 0.96 | 0.96 |
| Neur.Th.sd | 0.96 | 0.04 | 0.77 | 0.95 | 0.96 | 0.97 |
