# Supplementary material for: MicroCT-based phenomics in the zebrafish skeleton reveals virtues of deep phenotyping in a distributed organ system
Source: eLife. 2017 Sep 8;6:e26014. doi: 10.7554/eLife.26014 (PMC5606849; doi:10.7554/eLife.26014)
Supplement: Figure 7—source data 2. — Expected values for isometric scaling with standard length are as follows: Vol: b = 3, TMC: b = 3, Th: b = 1, Le: b = 1. [file elife-26014-fig7-data2.docx]

| Vert | Cent.Vol | Haem.Vol | Neur.Vol | Cent.TMC | Haem.TMC | Neur.TMC | Cent.Th | Haem.Th | Neur.Th | Cent.Le |
| --- | --- | --- | --- | --- | --- | --- | --- | --- | --- | --- |
| 1  2  3  4  5  6  7  8  9  10  11  12  13  14  15  16 | 2.36  2.26  2.38  2.35  2.41  2.45  2.47  2.46  2.52  2.54  2.62  2.68  2.57  2.61  2.54  2.52 | 2.52  2.65  2.80  2.79  2.96  3.04  3.32  3.08  2.84  2.88  2.28  2.76  2.74  3.17  3.12  3.37 | 2.23  2.48  2.60  2.90  3.34  3.37  3.67  4.00  4.06  3.93  4.37  4.05  3.76  3.43  3.51  3.63 | 4.57  4.50  4.57  4.56  4.66  4.71  4.78  4.72  4.76  4.75  4.85  4.86  4.72  4.76  4.72  4.73 | 4.56  4.84  5.06  5.05  5.25  5.32  5.61  5.44  5.29  5.12  4.59  5.28  5.25  5.82  5.64  5.96 | 4.64  4.87  5.04  5.37  6.01  6.04  6.85  6.92  6.75  7.23  7.27  6.87  6.55  6.06  6.22  6.45 | 0.31  0.37  0.34  0.33  0.34  0.38  0.40  0.31  0.32  0.30  0.39  0.37  0.28  0.30  0.35  0.26 | 0.50  0.50  0.60  0.60  0.63  0.54  0.53  0.47  0.50  0.29  0.46  0.71  0.66  0.97  0.84  0.82 | 0.49  0.33  0.40  0.44  0.39  0.35  0.31  0.51  0.61  0.55  0.67  0.72  0.74  0.73  0.81  0.81 | 0.95  0.92  1.10  1.07  1.07  1.07  1.11  1.10  1.07  1.14  1.14  1.17  1.09  1.17  1.02  1.14 |
|  |  |  |  |  |  |  |  |  |  |  |
